# Associations Between Sex‐ and APOE‐Specific Transcriptomic Signatures in Alzheimer's Disease and Imaging‐Derived Phenotypes: An AZ‐ADRC‐Research Education Scholars Team Science Project

**DOI:** 10.1002/alz.092738

**Published:** 2025-01-09

**Authors:** Adam C. Raikes, Francesca Vitali, Gerson D Hernandez, Fei Yin

**Affiliations:** ^1^ University of Arizona, Tucson, AZ USA

## Abstract

**Background:**

Age, female sex and the APOEε4 allele are among the top risk factors for developing late‐onset Alzheimer’s disease (LOAD). Precision medicine for AD drug development necessitates targeting specific biological pathways driving AD pathology. We previously identified LOAD‐associated transcriptomic signatures specific to both sex and APOE genotype. Here we extend these analyses to examine the association between these signatures and imaging‐derived phenotypes (IDPs).

**Method:**

Brain RNA‐Seq datasets from ROSMAP (syn8456637) were obtained from the RNA‐Seq Harmonization Study on AMP‐AD, including 369 frontal cortex samples from APOEε3/ε3 and APOEε3/ε4 individuals. Differentially expressed genes (DEGs, p‐value<0.01) between LOAD and cognitively normal controls were identified for each sex‐genotype. IDPs were generated from 1155 individuals in ROSMAP, with 62 overlapping the RNA‐Seq Harmonization Study. T1‐weighted MRIs were processed using Fastsurfer (v.2.0.4), and cortical thickness (CT) and subcortical volumes (SV) were computed. After multi‐site harmonization and covariate adjustment, effect sizes (Cohen’s d) for controls vs. AD were computed for each sex‐genotype pairing. Correlations between these effect sizes and median regional DEG expression from the Allen Human Brain Atlas were examined.

**Result:**

Controls showed anticipated CT and SV patterns, though male APOEε4s exhibited greater CT in more regions. Higher median expression of up (female APOEε3/ε3, male APOEε4) and down (female APOEε3/ε3, male APOEε3/ε3, male APOEε4) regulated DEGs correlated with less CT in AD patients (r=0.21‐0.41, p < 0.046). In APOEε4 carriers, higher median up (male, female) and down (female) DEG expression correlated with greater SV in AD patients (r=0.48‐0.57, p < 0.043). Greater median downregulated DEG expression in APOEε3/ε3s correlated with smaller SV (r=0.56, p=0.021). Higher median APOE expression was correlated with less CT in AD patients across sex‐genotype pairings (r=0.38‐0.86, p < 0.002), lower SV (females, all genotypes), and greater SV (male APOEε3/ε3). No associations between median DEG expression and CT were observed in female APOEε4 carriers.

**Conclusion:**

Our analyses reveal novel sex and APOE genotype‐specific transcriptomic signatures associations with imaging‐derived features in LOAD. Our findings transcriptome signature specific CT and SV profiles that may inform therapeutic targets. The present analyses provide support the identification and use of risk‐factor specific biomarkers of target engagement for preventative and therapeutic interventions.

